# Decoding Hearing-Related Changes in Older Adults’ Spatiotemporal Neural Processing of Speech Using Machine Learning

**DOI:** 10.3389/fnins.2020.00748

**Published:** 2020-07-16

**Authors:** Md Sultan Mahmud, Faruk Ahmed, Rakib Al-Fahad, Kazi Ashraf Moinuddin, Mohammed Yeasin, Claude Alain, Gavin M. Bidelman

**Affiliations:** ^1^Department of Electrical and Computer Engineering, The University of Memphis, Memphis, TN, United States; ^2^Rotman Research Institute–Baycrest Centre for Geriatric Care, Toronto, ON, Canada; ^3^Department of Psychology, University of Toronto, Toronto, ON, Canada; ^4^Institute of Medical Sciences, University of Toronto, Toronto, ON, Canada; ^5^Institute for Intelligent Systems, University of Memphis, Memphis, TN, United States; ^6^School of Communication Sciences and Disorders, University of Memphis, Memphis, TN, United States; ^7^Department of Anatomy and Neurobiology, University of Tennessee Health Science Center, Memphis, TN, United States

**Keywords:** speech perception, aging, event-related potentials, hearing loss, machine learning, stability selection and control, support vector machine

## Abstract

Speech perception in noisy environments depends on complex interactions between sensory and cognitive systems. In older adults, such interactions may be affected, especially in those individuals who have more severe age-related hearing loss. Using a data-driven approach, we assessed the temporal (*when* in time) and spatial (*where* in the brain) characteristics of cortical speech-evoked responses that distinguish older adults with or without mild hearing loss. We performed source analyses to estimate cortical surface signals from the EEG recordings during a phoneme discrimination task conducted under clear and noise-degraded conditions. We computed source-level ERPs (i.e., mean activation within each ROI) from each of the 68 ROIs of the Desikan-Killiany (DK) atlas, averaged over a randomly chosen 100 trials without replacement to form feature vectors. We adopted a multivariate feature selection method called stability selection and control to choose features that are consistent over a range of model parameters. We use parameter optimized support vector machine (SVM) as a classifiers to investigate the *time course* and *brain regions* that segregate groups and speech clarity. For clear speech perception, whole-brain data revealed a classification accuracy of 81.50% [area under the curve (AUC) 80.73%; F1-score 82.00%], distinguishing groups within ∼60 ms after speech onset (i.e., as early as the P1 wave). We observed lower accuracy of 78.12% [AUC 77.64%; F1-score 78.00%] and delayed classification performance when speech was embedded in noise, with group segregation at 80 ms. Separate analysis using left (LH) and right hemisphere (RH) regions showed that LH speech activity was better at distinguishing hearing groups than activity measured in the RH. Moreover, stability selection analysis identified 12 brain regions (among 1428 total spatiotemporal features from 68 regions) where source activity segregated groups with >80% accuracy (clear speech); whereas 16 regions were critical for noise-degraded speech to achieve a comparable level of group segregation (78.7% accuracy). Our results identify critical time-courses and brain regions that distinguish mild hearing loss from normal hearing in older adults and confirm a larger number of active areas, particularly in RH, when processing noise-degraded speech information.

## Introduction

Hearing impairment (HI) is the fifth leading disability worldwide (Vos et al., 2015) and the third most common chronic disease behind heart disease and arthritis (Blackwell et al., 2014; Liberman, 2017). It is one of the core contributors to the growing disability problem in the United States (Murray et al., 2013). In older adults, HI has been associated with poor cognitive health, social isolation, and loneliness (Lin et al., 2013; Diaz et al., 2018). Speech processing in the elderly relies on a complex network of interacting brain regions (Hickok and Poeppel, 2004, 2007; Rauschecker and Scott, 2009; Bidelman et al., 2019a). Age-related HI is thought to occur due to a myriad of changes in both the central auditory pathways (Bidelman et al., 2014, 2019b) and widespread areas of both cerebral hemispheres (Gates and Mills, 2005). For example, studies have shown aged-related declines in the temporal precision (Roque et al., 2019) of (subcortical) neural encoding (Anderson et al., 2012; Konrad-Martin et al., 2012; Bidelman et al., 2014; Schoof and Rosen, 2016) and functional magnetic resonance imaging (fMRI) has shown older adults have greater activation than younger adults in widespread cortical brain regions (Du et al., 2016; Mudar and Husain, 2016; Diaz et al., 2018). Older adults with hearing impairment show even greater activation in right hemisphere (RH) than the left hemisphere (LH) during speech perception in noise (Mudar and Husain, 2016). Similarly, the hemispheric asymmetry reduction in older adults (HAROLD) model (Cabeza, 2002) posits that older adults show a reduction in hemispheric asymmetry during episodic encoding and semantic retrieval.

Speech-in-noise (SIN) perception can be difficult for older adults, especially in those with hearing loss. The neurophysiological factors that influence SIN perception are not fully understood, but likely involve rapid temporal processing. As such, tracking the neural encoding of speech necessitates use of neuroimaging techniques with excellent temporal resolution, such as event-related potentials (ERPs). EEG/ERPs also offer a non-invasive means for clinical diagnostics, including those related to cognitive aging as well as tracking how the brain encodes important features of the speech signal (Bidelman et al., 2017). For instance, the auditory cortical ERPs, comprised of the P1, N1, and P2 waves, are highly sensitive to the acoustic features of speech (Agung et al., 2006), and correlate with listeners’ perception of both clear and noise-degraded speech (Tremblay et al., 2001; Ross et al., 2009; Bidelman et al., 2014).

Evidence suggests that older adults incorporate more attentional resources than younger adults in auditory perceptual tasks (Alain et al., 2004; Grady, 2008). This could account for some of the age-related increases in ERP amplitudes reported in HI vs. normal hearing (NH) listeners (Alain, 2014; Bidelman et al., 2019b). Prior neuroimaging studies (Peelle et al., 2009; Wong et al., 2009; Erb and Obleser, 2013; Vaden et al., 2015) have also demonstrated increased activity in prefrontal regions related to cognitive control, attention, and working memory when older listeners process speech under challenging situations. In noisy environments, left inferior frontal gyrus and left inferior parietal lobe are recruited for speech perception (Dimitrijevic et al., 2019). In our earlier EEG studies (Bidelman et al., 2019a; Price et al., 2019), we used functional connectivity analysis to demonstrate that older listeners with mild hearing loss had more extended (less integrated) communication pathways and less efficient information exchange across the brain than their NH peers; directed connectivity analysis further showed that age-related HI reverses the direction of neural signaling within important hubs of the auditory-linguistic-motor loop of the dorsal-ventral pathways (e.g., primary auditory cortex – inferior frontal gyrus – primary motor cortex), implying a rerouting of information within the same speech circuitry (Bidelman et al., 2019a).

Our previous study focused on a restricted set of speech-relevant brain regions compared to the widespread and distributed networks involved in speech-language function (Rauschecker and Scott, 2009; Du et al., 2014, 2016). Machine learning can predict and identify subtle changes in neural activity very accurately and quickly, without intervention from human observers. It would be meaningful if brain function related to hearing loss in older adults could be identified from neural data without *a priori* assumptions. Extending prior hypothesis-driven work on the aging brain, here, we take an entirely different, comprehensive data-driven approach to test whether hearing status can be decoded from full-brain activity based on how listeners process speech. We aimed to identify the most probable global set of brain regions that are sensitive to HI in older adults. To our knowledge, this is the first study to apply decoding and machine learning techniques to map spatiotemporal differences in speech and SIN processing in older listeners at the full-brain level.

The current study aimed to investigate neural changes associated with HI on full-brain functionality using a data driven multivariate approach (machine learning). We applied source analysis to scalp-recorded electrical brain activity recorded in older adults while they were presented with clear or noise-degraded speech. ERPs were expected to differ for noise-degraded compared to clear speech due to a reduction of neural synchrony (Koerner and Zhang, 2015) and more widespread engagement of neural resources in challenging acoustics (Brette, 2012; Kim et al., 2012), including right hemisphere (Bidelman and Howell, 2016). We also anticipated more dramatic group differences in noise since older adults with mild hearing loss are most challenged in degraded listening conditions (Tremblay et al., 2003).

We hypothesized that speech-evoked responses among NH and HI listeners would differ with regards to time and spatial regions that are recruited during phoneme discrimination. We further hypothesized that the speech network “decoded” via machine learning would vary as a function of the clarity of the speech signal. We applied a data-driven approach (support vector machine (SVM), stability selection) to source-level EEG data to identify when (*in time*) and where *(brain regions of interest, ROIs)* hearing status could be decoded from brain activity. We have recently used a similar approach to decode perceptual decisions from the EEG during speech categorization tasks (Al-Fahad et al., 2019). We used a sliding window decoder to address the temporal dynamics of decoding and identify when speech-evoked responses distinguished older adults with NH or mild hearing loss. In addition, stability selection, a machine learning approach to identify highly consistent data features, was used to examine where in the brain group responses best separated older adults with NH vs. mild hearing loss.

## Materials and Methods

Analyses of the ERPs and behavioral data associated with this dataset are reported elsewhere (Bidelman et al., 2019a, b). In this study, we present a new machine learning analysis to identify the most discriminating spatiotemporal features of full-brain neuroelectric activity that best segregates NH and mild hearing loss listeners in terms of their SIN processing. We perform group analysis (NH vs. HI) in both conditions (clear and noise) using source-derived neural activity. We used neural data as the input to classifiers (e.g., SVM) to identify when in time group segregation was best. Furthermore, we used a robust variable selection technique called stability selection to find brain regions that are associated with hearing loss.

### Participants

**FIGURE 1:**
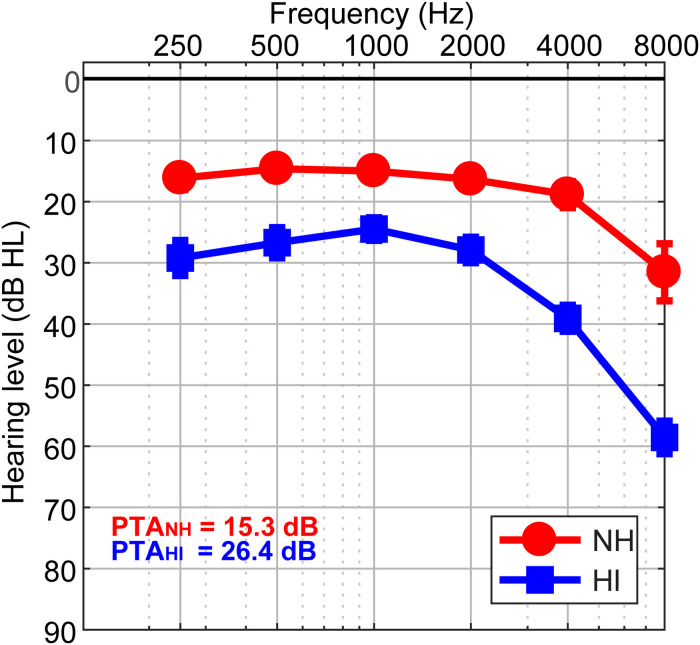
Behavioral audiograms (hearing thresholds) per group. NH, normal-hearing listeners; HI, mild hearing loss listeners; PTA, puretone average threshold.

The sample consisted of thirty-two older adults (13 NH and 19 HI; aged 52–72 years). Demographic details are provided in our previous reports (Mahmud et al., 2018; Bidelman et al., 2019a, b). The range of average hearing thresholds for the HI cohort was (25.83 dB to 49.16 dB) and the NH cohort was (12.08 dB to 23.75 dB) across both ears. Listeners were divided into two cohorts based on their average hearing thresholds being better (NH) or poorer (HI) than 25 dB HL across both ears (Figure 1). The groups were matched in age (NH: 66.2 ± 6.1 years, HI: 70.4 ± 4.9 years; *t*_22.2_ = −2.05, *p* = 0.052) and gender balance (NH: 5/8 male/female; HI: 11/8; Fisher’s exact test, *p* = 0.47). Puretone average thresholds between ears was symmetric in both the NH [*t*_12_ = 0.15, *p* = 0.89] and HI [*t*_1__8_ = −2.02, *p* = 0.06] groups. Age and hearing loss were not correlated (Pearson’s *r* = 0.29, *p* = 0.10). No cognitive function was screened. All originally gave written informed consent in accordance with a protocol approved by the Baycrest Research Ethics Review Board.

### Stimuli and Task

Speech tokens (/ba/, /pa/, and /ta/; 100 ms duration) (Dubno and Schaefer, 1992) were presented back-to-back in random order with a jittered interstimulus interval (95–155 ms). For both the clear (i.e., no noise) and noise conditions, the stimulus set included a total of 3000 /ba/, 3000 /pa/, and 210 /ta/ tokens (spread evenly over three blocks to allow for breaks. Frequent (/ba/, /pa/) and infrequent (/ta/) tokens were presented according to a pseudo-random schedule where at least two frequent stimuli intervened between target /ta/tokens. Listeners were asked to detect target /ta/ tokens. For noise blocks, the speech triplet was mixed with noise babble (Killion et al., 2004) at 10 dB signal to noise ratio (SNR). Stimuli were presented binaurally at 75 dB_A_ sound pressure level (SPL) (noise at 65 dB_A_ SPL)^1^. The task lasted ∼20 min. Analysis of the behavioral data associated with this task are reported elsewhere (Bidelman et al., 2019a).

### EEG Recording

During the behavioral task, neuroelectric activity was recorded from 32 channels at standard 10–20 electrode locations on the scalp (Oostenveld and Praamstra, 2001). Preprocessing procedures followed our published reports (Bidelman et al., 2019a, b). Data were re-referenced off-line to a common average. Following ocular artifact correction (Picton et al., 2000) cleaned EEGs were then filtered (1–100 Hz; notched filter 60 Hz), epoched (−10–200 ms), and averaged in the time domain (described in section “Feature Extraction”) to derive ERPs for each stimulus condition per participant. Responses to non-targets (/ba/ and /pa/ tokens) were collapsed to reduce the dimensionality of the data. Infrequent /ta/ responses were not analyzed given their limited number of trials.

### EEG Source Localization

We localized the sources of the scalp-EEG data by performing a distributed source analysis. We performed source localization in the MATLAB package Brainstorm (Tadel et al., 2011) using a realistic boundary element head model (BEM) volume conductor (Fuchs et al., 1998, 2002) standardized to the MNI template brain (Mazziotta et al., 1995) – used for all participants. The BEM head model was implemented using the OpenMEEG module in Brainstorm (Gramfort et al., 2010). A BEM is less prone to spatial errors than other existing head models (e.g., concentric spherical conductor) (Fuchs et al., 2002). Essentially, the BEM model parcellates the cortical surface into 15,000 vertices and assigns a dipole at each vertex with an orientation perpendicular to the cortical surface. From the pre-stimulus interval, the noise covariance matrix was estimated. We then used standard low-resolution brain electromagnetic tomography (sLORETA) to create inverse solutions (Pascual-Marqui et al., 2002). We used Brainstorm’s default regularization parameters (regularized noise covariance = 0.1; SNR = 3.00). sLORETA provides a smoothness constraint that ensures the estimated current changes little between neighboring neural populations on the brain (Picton et al., 1999; Michel et al., 2004). Localization of sLORETA for 32 channel is ∼1.5x less accurate than 64 channels (Michel et al., 2004). Nevertheless, mean localization error for sLORETA is estimated to be 1.45 mm for 32 channels (Song et al., 2015). An important point is that these methods were applied uniformly across all listeners/groups. Thus, while overall source locations might be underestimated in our source reconstruction, this would not account for group differences. From each single trial sLORETA brain volume, we extracted the time-course of source activity within regions of interest (ROI) defined by the Desikan-Killany (DK) atlas parcellation (Desikan et al., 2006). This atlas has 68 ROIs (e.g., LH: 34 ROIs, and RH: 34 ROIs). Subsequently, these time-courses were baseline corrected from the pre-stimulus interval and then used as input to the SVM and stability selection to investigate *when* and *where* brain activity distinguishes NH and HI groups.

### Feature Extraction

Generally, averaging over more trials enhances the SNR of the ERPs by reducing EEG noise. Our dataset included ∼6000 trials per participant and per condition (clear, noise) that can provide an adequate number of training and test examples using ERPs computed with different subsets of trials (without replacement). From each of the 68 ROIs of the DK atlas, we extracted source-level ERPs (i.e., mean activation within each ROI) averaged over randomly chosen 25, 50, 75, 100, and 125 trials without replacement. We then analyzed the ERP time courses using a sliding window basis (10 ms without overlap) across the whole epoch. Empirically, we found that responses averaged over 100 trials yielded the best classification results, providing a balance between classifier performance, computational speed, while also ensuring adequate SNR of the ERPs. 100 trial averages are therefore reported hereafter. The sliding window resulted in 21 (i.e., 210/10 ms) ERP features (i.e., mean amplitude per window) for each ROI waveform, yielding 68^∗^21 = 1428 features for each condition (e.g., clear and noise). These features were used as the input to the SVM classifier and stability selection coupled with SVM framework. As is common in classifiers, data were z-score normalized prior to classification and stability selection in order to ensure all features were on a common scale and range (Casale et al., 2008).

### SVM Classification

Data driven multivariate analysis are a mainstay in modeling complex data and understand the relationship among all possible variables. Parameter optimized SVM classifiers are better candidate in building robust discriminative models with small sample sizes, which is common in human neuroimaging studies (Furey et al., 2000; Polat and Güneş, 2007). Classifier performance is greatly affected by the choice of kernel function, which can be used to map nonlinearly separable data to linearly separable space. Other tunable parameters (e.g., kernel, *C*, γ)^2^ also alter performance (Hsu et al., 2003). As such, we used a grid search approach (range of *C* = 1e-2 to 1e2, and γ = 1e-2 to 7e-4) to find the optimal kernel, *C*, and γ values (kernels = ‘RBF’). We randomly split the data into training and test sets (80% and 20%, respectively) (Park et al., 2011).

During the training phase, we fine-tuned the *C* and γ parameters to find optimal values for the classifier that maximally distinguished observations from the NH vs. HI group. The SVM learned the support vectors from the training data that comprised the attributes (e.g., ERPs amplitudes) and class labels (e.g., NH and HI). The resulting hyperplanes were fixed with maximum margin (e.g., maximum separation between the two classes) and used for predicting the unseen test data (by providing only the attributes but no class labels). Classification performance was calculated from standard formulas (accuracy, *F*1-score, and area under the curve (AUC)) (Saito and Rehmsmeier, 2015). AUC is a discriminating metric that describes the degree to which the model is capable of distinguishing between classes. An excellent model has AUC near to 1, meaning it has a good measure of separability. On the other hand, a poor model has AUC close to 0, meaning it has poor separability.

### Stable Feature Selection (Stability Selection)

A robust model should be complete enough to allow generalization and be easy to interpret. On the contrary, large numbers of feature variables (several thousand, as measured here) are susceptible to overfitting and can lead to models that lack interpretability. This requires selecting a set of the most salient discriminating features that are consistent across a range of model parameters. Feature selection is difficult, especially when the number of samples are small as compared to the number of features. Stability selection is a state-of-the-art feature selection method that works well in high dimensional or sparse problems based on Lasso (least absolute shrinkage and selection operator) (Meinshausen and Bühlmann, 2010; Yin et al., 2017). Stability selection uses a Randomized Lasso algorithm, which works by resampling the training data set and computing feature scores on each resampling over the range of regularization parameters. Because stability selection includes an internal randomization technique (over many interactions), it yields a more reliable and consistent feature set than the conventional filtering and other multivariate approaches. Stability selection can identify the most stable (relevant) features from a large number of features, even if the necessary conditions required for the original Lasso method are violated (Meinshausen and Bühlmann, 2010).

In stability selection, a feature is considered to be more stable if it is more frequently selected over repeated subsampling of the data (Nogueira et al., 2017). Basically, the Randomized Lasso randomly subsamples the training data and fits a L1-penalized logistic regression model to optimize the error. Lasso reduces the variance without substantially increasing bias during the subsampling process. Over many iterations, feature scores are (re)calculated. The features are shrunk to zero by multiplying the features’ coefficient by zero while the stability score is lowered. Surviving non-zero features are considered important variables for classification. Detailed interpretation and mathematical equations of stability selection are explained in Meinshausen and Bühlmann (2010). The stability selection solution is less affected by the choice of initial regularization parameters. Consequently, it is extremely general and widely used in high dimensional data even when noise level in the data is unknown.

We considered sample fraction = 0.75, number of resamples = 1000, with tolerance = 0.01 (Meinshausen and Bühlmann, 2010; Al-Fahad et al., 2019) in our implementation of stability selection^3^. In the Lasso algorithm, the feature scores were scaled between 0 and 1, where 0 is the lowest score (i.e., irrelevant) and 1 is the highest score (i.e., most salient or stable feature). We estimated the regularization parameter from the data using the least angle regression (LARs) algorithm (Efron et al., 2004; Friedman et al., 2010). Over 1000 iterations, Randomized Lasso provided the overall feature scores (0∼1) based on frequency a variable was selected. We ranked stability scores to identify the most important, consistent, stable, and invariant features (i.e., neural amplitudes across all ROIs and time) over a range of model parameters. We submitted these ranked features and corresponding class labels to an SVM classifier. Based on the input stable features, SVM classified the group membership with different stability threshold values. The stability threshold corresponding to the model that yielded maximum accuracy, AUC, and F1-score was considered as the optimal threshold.

## Results

### ERPs in HI vs. NH Older Adults

**FIGURE 2:**
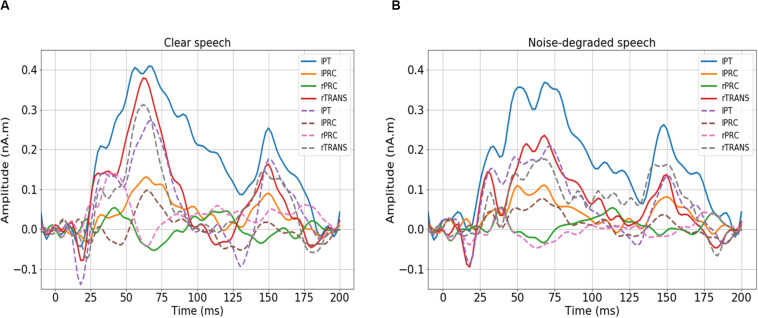
Source-level ERPs for the NH and HI groups in representative ROIs. Solid lines = HI; dotted lines = NH. **(A)** Clear speech responses. **(B)** Noise-degraded speech responses. Baseline was corrected to the prestimulus interval. NH, normal hearing; HI, hearing impaired; L, Left; R, Right; lPT, parstriangularis L; lPRC, precentral L; rPRC, precentral R; rTRANS, transverse temporal R.

We first visualized the source (region-specific) ERPs of NH and HI listeners during clear and noise-degraded speech perception. Figure 2 presents source waveforms for clear and noise-degraded speech within four representative ROIs among the auditory-linguistic-motor loop that are known to be important in speech processing in older listeners (Bidelman et al., 2019a): primary auditory cortex [transverse temporal (TRANS) gyrus], primary motor cortex [precentral (PRC) gyrus], Broca’s area [paras triangularis (PT)]. In both conditions (clear and noise-degraded) HI generally showed higher ERPs than NH (Figure 2). A detailed analysis of the ERPs is reported elsewhere (Bidelman et al., 2019b).

### SVM Classification of Hearing Status Using ERP Features

**FIGURE 3:**
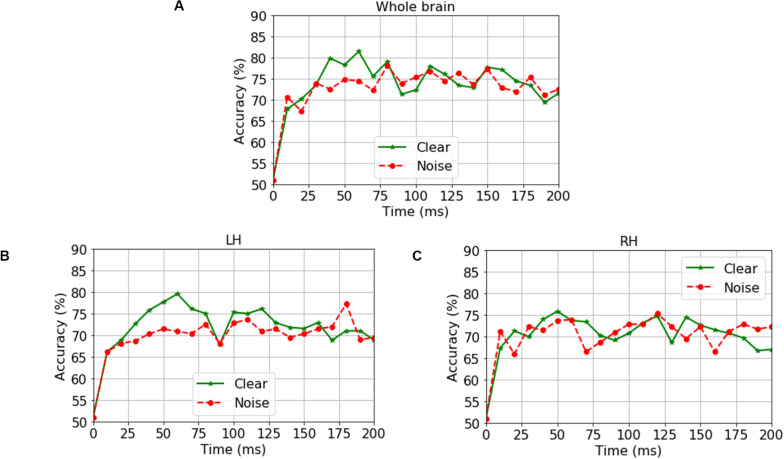
Time-varying group classification (NH vs. HI) as a function of neural data (clear and noise conditions) and hemisphere. Group classification accuracy from **(A)** Whole-brain data (all 68 ROIs), **(B)** LH data alone (34 ROIs), and **(C)** RH data alone (34 ROIs). LH, left hemisphere; RH, right hemisphere. 0 ms = stimulus onset. Green solid line indicates group segregation during clear speech perception, red dotted line indicates group segregation during noise-degraded speech perception.

**FIGURE 4:**
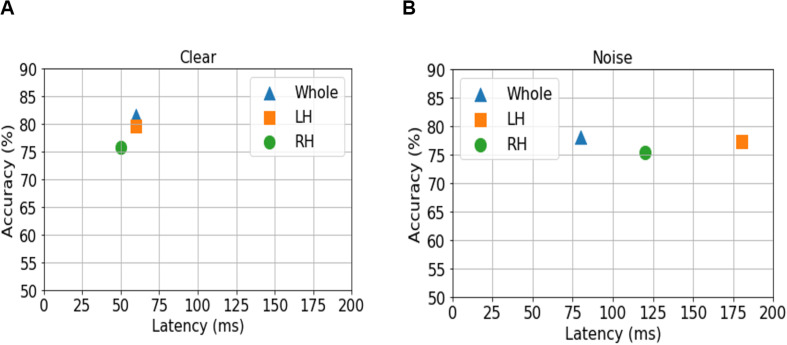
Maximum classifier accuracy (*y* axis) and corresponding latency (*x* axis) for distinguishing NH and HI listeners using source amplitudes from the whole-brain (blue triangle), and LH (orange square) vs. RH (green circle) separately. **(A)** Clear speech responses. **(B)** Noise-degraded speech responses.

We analyzed group classification performance using (i) whole-brain source waveform data and (ii) each hemisphere (e.g., LH and RH) individually. We submitted ERP amplitudes and corresponding class labels to the SVM using a sliding window (10 ms) basis over the entire 210 ms epoch window (see Figure 2). We used 5-fold cross-validation^4^ (Bhasin and Raghava, 2004) and carried out the grid search approach during the training period to determine the optimal parameters of the classifier. The optimal values of C and γ parameters corresponding to the maximum group segregation reported in Table 1 were: [clear speech: *C* = 20, γ = 0.01 for whole-brain data; *C* = 70, γ = 0.01 for LH; and *C* = 70, γ = 0.01 for RH. Noise-degraded speech: *C* = 10, γ = 0.01 for whole-brain data; *C* = 30, γ = 0.01 for LH; and *C* = 30, γ = 0.01 for RH]. We then selected the best model and performance metrics from the predicted class labels, which were obtained from the unseen test data as well as true class labels. We applied the SVM classifier on features extracted over 10 ms sliding windows to categorize NH vs. mild hearing loss. The accuracy over each time window is presented in Figure 3; maximum accuracy and its corresponding latency are shown in Figure 4 and summarized in Table 1.

**TABLE 1:** SVM classifier maximum performance (%) distinguishing hearing status (NH vs. HI).

Speech stimulus	Measure	Whole brain features	LH features	RH features
Clear	Accuracy	81.50	79.62	75.87
	AUC	80.73	79.75	75.25
	F1-score	82.00	80.00	76.00
Noise	Accuracy	78.12	77.28	75.34
	AUC	77.64	76.93	75.19
	F1-score	78.00	77.00	75.00

*Maximum classification based on the time-varying SVM results shown in Figure 3. AUC, area under the receiver operating characteristic (ROC) curve. F1-score = 2 (precision × recall)/(precision +recall). Chance level is 50%. LH: left hemisphere; RH: right hemisphere.*

For ERP responses elicited by *clear* speech, the classifier conducted on full-brain data (all 68 ROIs) yielded a maximum accuracy in labeling groups of 81.50% at 60 ms. Classification was still quite accurate using LH (34 ROIs) responses alone (79.62%), but group segregation occurred at the same latency at 60 ms. The poorest accuracy was obtained using RH (34 ROIs) features alone (75.87%) at a latency of 50 ms.

For ERP responses to *noise*-*degraded* speech, maximum classification occurred later than for clear speech. The maximum classifier accuracy was 78.12% at 80 ms using full-brain data. The LH features showed slightly lower accuracy (77.28%) than the whole brain at a latency of 180 ms. RH features provided the lowest accuracy (75.34% at 120 ms), among the three feature scenarios. Still, these group classification results are well above chance (i.e., 50%) and reveal the temporal dynamics of cortical speech activity that robustly identifies older listeners with mild hearing loss.

### Stability Selection Coupled With SVM

**FIGURE 5:**
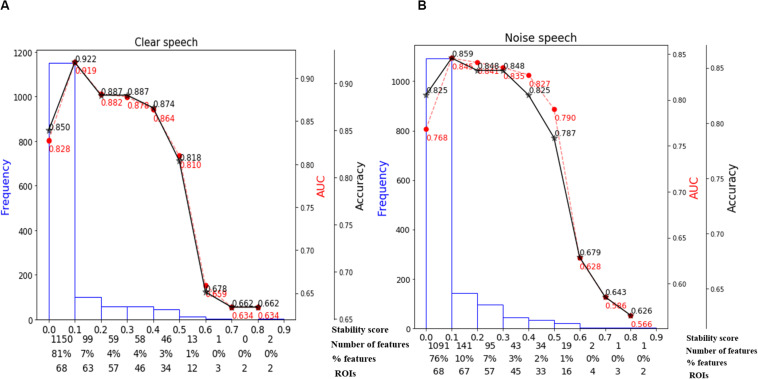
Effect of stability score threshold on model performance. The bottom of *x*-axis has four labels; *Stability score*, represents the stability score range of each bin (scores: 0∼1); *Number of features*, number of features under each bin; *% features*, corresponding percentage of selected features; *ROIs*, number of cumulative unique brain regions up to lower boundary of the bin. **(A)** Clear speech. **(B)** Noise-degraded speech.

We used stability selection to identify the most important brain ROIs that segregate groups without overfitting. ERP amplitude features were considered stable if they yielded higher stability scores at an 80% criterion level of classification performance (i.e., >80% group separation). During pilot modeling, we roved stability thresholds, which yielded different levels of classification performance. The effect of stability selection threshold on model performance is delineated in Figure 5A (clear) and Figure 5B (noise-degraded). The histogram shows the distribution of feature scores. The first line of *x*-axis represents the stability score (0 to 1); the second and third line represent the number and percentage of selected features under the corresponding bin; line four shows the number of cumulative unique brain ROIs up to the lower boundary of the bin. The semi bell-shaped solid black and red dotted lines of Figure 5 indicate the accuracy and AUC curve for different stability scores, respectively. In our stability selection analysis, the number of features represents ROI-specific source ERP amplitudes (in different time windows) and the number of unique ROIs represent functionally distinct brain areas of the DK atlas.

The selected subset of features from the whole-brain identified via stability selection were then submitted to an SVM. For both clear and noise-degraded speech, the SVM classifier performance changed with the choice of stability threshold. We found that for clear speech, 81% of the features had scores (0 to 0.1) whereas 76% for noise-degraded speech detection. This means that the majority of ERP features were selected <10% of the time out of 1000 model iterations and thus carried near-zero importance in terms of segregating groups. Thus, 81% of the features were not related to segregating groups for clear speech, and 76% were irrelevant features for noise-degraded conditions.

For clear speech, maximum accuracy in distinguishing groups (92.2% accuracy; AUC 91.9%, F1-score 92.0%) was achieved using a stability score threshold of 0.10. At this threshold, the number of selected features was 278 (19%) out of 1428 from the 68 ROIs. For noise-degraded speech, stability selection selected 337 (24%) out of 1428 features from 68 ROIs, corresponding to 85.9% accuracy (AUC 84.5%, F1-score 86.0%). Less than or greater than this optimal stability threshold the classifier showed poorer performance. Below the optimal threshold of 0.1, classifier performance was lower because irrelevant features were selected, whereas above the 0.1 threshold, some relevant features for distinguishing hearing status were discarded.

Moreover, even when we selected a stability threshold of 0.7 (more conservative feature selection), clear speech responses could segregate groups with 66.2% accuracy using only two ROIs (top two in Table 2). In contrast, noise-degraded speech yielded 64.7% accuracy with only three ROIs. These results indicate that hearing status can be decoded still above chance levels using only a few brain regions engaged during speech perception. It is also notable that a larger number of ROIs were selected in noise-degraded speech perception as compared to clear speech perception corresponding to the same stability threshold and accuracy.

**TABLE 2:** Most important brain regions (clear: 12 ROIs; noise: 16 ROIs; 0.50 stability threshold) distinguishing age-related hearing loss via EEG.

Rank	Clear (81.8% total accuracy)			Noise (78.7% total accuracy)		
	ROI name	ROI abbrev.	Stability score	ROI name	ROI abbrev.	Stability score
1	Temporal pole R	rTP	0.86^a^	Rostral middle frontal L	lRMF	0.93
2	Fusiform R	rFUS	0.84	Fusiform R	rFUS	0.81
3	Superior parietal L	lSP	0.65	Inferior temporal R	rIT	0.7
4	Precentral R	rPRC	0.60	Caudal middle frontal R	rCMF	0.64
5	Precentral L	lPRC	0.58	Inferior parietal L	lIP	0.60
6	Caudal middle frontal R	rCMF	0.55	Bankssts R	rBKS	0.60
7	Precuneus L	lPREC	0.55	Paracentral R	rPARAC	0.58
8	Middle temporal L	lMT	0.53	Precentral R	rPRC	0.57
9	Isthmus cingulate R	rIST	0.53	Bankssts L	lBKS	0.57
10	Bankssts L	lBKS	0.52	Temporal pole R	rTP	0.55
11	Bankssts R	rBKS	0.51	Para hippocampal L	lPHIP	0.53
12	Superior temporal L	lST	0.50	Isthmus cingulate R	rIST	0.53
13	–	–	–	Superior temporal R	rST	0.53
14		–	–	Pericalcarine L	lPERI	0.52
15	–	–	–	Superior parietal L	lSP	0.51
16	–	–	–	Inferior parietal R	rIP	0.50

*^*a*^ Here a score of 0.86, for example, means that out of 1000 iterations, the ERP feature of this ROI was selected 860 times by stability selection.*

**FIGURE 6:**
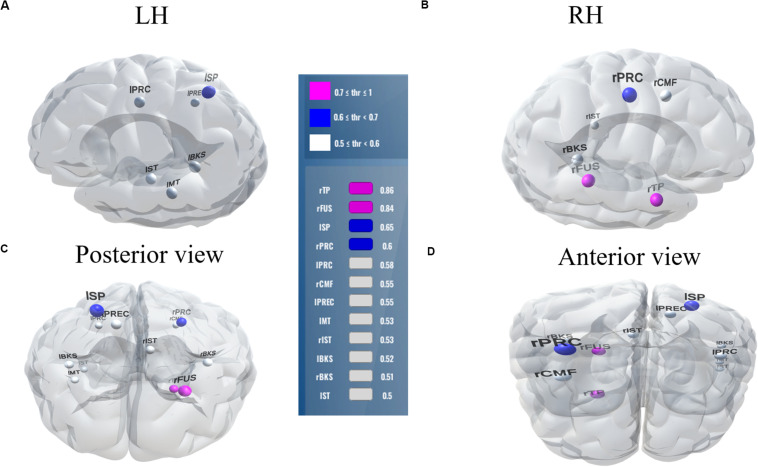
Stable (most consistent) neural network distinguishing NH and HI listeners during *clear* speech processing. Visualization of brain ROIs corresponding to 0.50 stability threshold (12 top selected ROIs which segregate groups at 81.8%) for clear speech perception. **(A)** LH; **(B)** RH; **(C)** Posterior view; **(D)** Anterior view. Stability score (color legend): (0.70 ≤ pink ≤ 1.0); (0.60 ≤ blue < 0.70); (0.50 ≤ white < 0.60). L, Left; R, Right; rTP, temporal pole R; rFUS, fusiform; lSP, sperior parietal L; rPRC, precentral R; lPRC, precentral L; rCMF, caudal middle frontal R; lPREC, precuneus L; lMT, middle temporal L; rIST, isthmuscingulate R; lBKS, bankssts L; rBKS, bankssts R; lST, superior temporal L.

**FIGURE 7:**
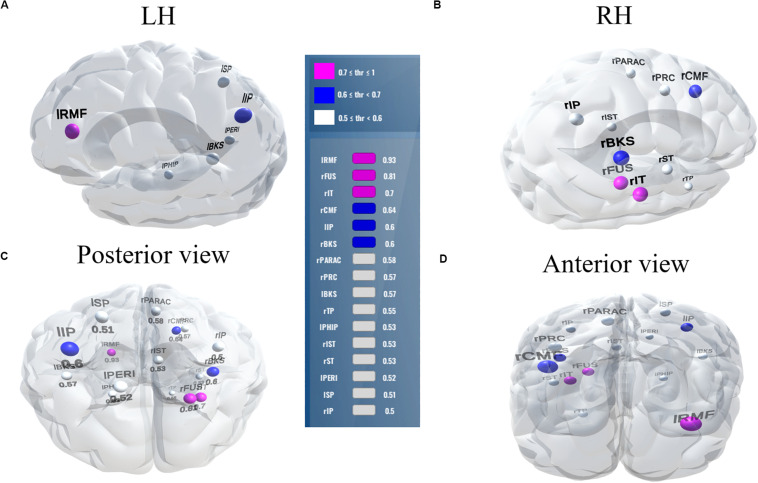
Stable (most consistent) neural network that distinguishes NH and HI listeners during *noise-degraded* speech processing. 16 top selected ROIs, 78.7% group classification. **(A)** LH; **(B)** RH; **(C)** Posterior view; **(D)** Anterior view. lRMF, rostral middle frontal L; rIT, inferior temporal R; lIP, inferior parietal L; rPARAC, paracentral R; lPHIP, para hippocampal L; rST, superior temporal R; lPERI, pericalcarine L; rIP, inferior parietal R. Otherwise as in Figure 6.

Balancing these lax vs. strict models, we found that for clear speech, a mid-level stability threshold of 0.5 segregated groups at 81.8% accuracy [AUC (81.0%) and F1-score (82.0%)] by selecting only 16 features from 12 unique ROIs (Table 2). Accuracy degraded by ∼10% (92.2% to 81.8 %) from the optimal value but the number of features reduced dramatically from 278 to 16, stemming from only 12 (rather than 63) ROIs. For noise-degraded speech perception, only 24 features were selected from 16 unique ROIs and produced accuracy 78.7%, AUC 79.0% and F1-score 79.0% [i.e., accuracy degraded by 7% from optimal accuracy (85.9%)] but well above chance level even in noise-degraded conditions. Thus, we considered a stability selection threshold of 0.5 which provided reasonable performance and less computation, but more critically, an interpretable network to describe neurophysiological speech processing. The network of brain ROIs (at 0.5 stability threshold) for clear and noise-degraded speech perception are shown in Figures 6 and 7 using the BrainO (Moinuddin et al., 2019) visualization tool. Additional details are provided in Table 2.

## Discussion

In this study, we performed multivariate analyses on EEG to decode the spatiotemporal dynamics of neural speech processing and identify *when* and *where* brain activity is most sensitive to age-related declines in hearing.

### Hearing Status Is Decoded Early Within the Time-Course of Speech Processing

Our data corroborate previous studies showing speech-ERPs are higher in amplitude for clear compared to noise-degraded speech detection and HI compared to NH listeners, consistent with the effects of hearing loss and background noise on auditory cortical responses (Alain, 2014; Bidelman et al., 2014, 2019b). Extending previous work, we used these ERP attributes in SVM classification to assess the time-course of the brain’s response to speech and their ability to segregate normal and hearing-impaired listeners. Among the three classification scenarios (e.g., whole-brain, LH, and RH), whole-brain data provided the best group differentiation in the time frame of the P1 wave (∼50 ms). Additionally, LH activity provided better differentiation of groups’ speech processing than RH. Improved group discrimination in the timeframe of the P1 is consistent with previous reports, which show abnormally large P1 responses in older adults with HL (Woods and Clayworth, 1986; Tremblay et al., 2003; Snyder and Alain, 2005; Alain and Snyder, 2008; Bidelman et al., 2014; Zendel and Alain, 2014; Bidelman and Walker, 2019). Increased P1, a wave reflecting the early registration of sound in auditory cortex, presumably results from deceased neural inhibition (Caspary et al., 2008) and/or increased deafferentation (Kujawa and Liberman, 2015) that accompanies aging.

Classification based on the sliding window analysis (full-brain level) showed maximum group decoding accuracy at 60 ms for clear speech, whereas noise-degraded speech, maximum accuracy was observed later at 80 ms. These results suggest that the P1 can be a useful attribute to segregate NH and HI listeners’ neurophysiological processing of speech but also depends on the clarity (i.e., SNR) of the speech signal. Since the P1 wave is generated by the thalamus and primary auditory cortex (Erwin and Buchwald, 1987; Liegeois-Chauvel et al., 1994; McGee and Kraus, 1996; Eggermont et al., 1997; Jang et al., 2010), this suggests mild hearing loss in older adults changes auditory processing in early sensory regions. Furthermore, for noise-degraded speech, maximum group segregation was delayed relative to clear speech using whole-brain, LH and RH data. These delays were 20 ms for whole-brain, 120 ms for LH, and 70 ms for RH relative to clear speech. The later decoding for noise-degraded speech is perhaps expected due to inherent masking of the stimulus signal, which weakens the neural representation for speech, decreases the amplitude, and prolongs the latency of the ERPs. Previous studies have indeed shown that neural responses are significantly influenced by noise at the level of the midbrain (Burkard and Sims, 2002; Anderson et al., 2010; Ding and Simon, 2013; Presacco et al., 2016) and cortex (Billings et al., 2013, 2015; Bidelman and Howell, 2016; Bidelman and Yellamsetty, 2017). Thus, the delay in maximum decoding accuracy we find in the SIN condition is consistent with prior work. Moreover, the better performance by LH compared to RH activity in distinguishing groups is consistent with the dominance of LH in phoneme discrimination and speech sound processing (Zatorre et al., 1992; Frost et al., 1999; Tervaniemi and Hugdahl, 2003; Bidelman and Howell, 2016; Bidelman and Walker, 2019).

Our data cannot adjudicate the *cause* of older adults’ hearing-related changes in the EEG. Hearing loss is defined clinically by the audiogram, which is thought predominately to reflect peripheral (cochlear) integrity (Kujawa and Liberman, 2015). In addition to peripheral damage, changes in the central auditory pathway (Caspary et al., 2006; Humes et al., 2012; Bidelman et al., 2014), decreased gray and white matter in the central nervous system (Peelle and Wingfield, 2016), and eventually age-related atrophy that limits cognitive capacities all contribute to hearing issues in older adults (Humes et al., 2013). Regardless of the underlying etiology, it is clear that hearing-related changes manifest in neural reorganization, which is decodable in cortical scalp potentials. Moreover, our sample included listeners with relatively homogenous hearing impairment (less than mild HL) and we did not screen for cognitive function. Although we adopt widely used clinic criteria, there is typically large individual differences in older adults speech perception even with a similar (or even normal) audiogram (Konkle et al., 1977; van Rooij and Plomp, 1992; Gordon-Salant and Fitzgibbons, 1993; Cruickshanks et al., 1998; Strouse et al., 1998; Schneider et al., 2002; Hutka et al., 2013). However, behaviorally, our older HI listeners showed little decrement in speech detection accuracy relative to their NH peers, although they were more variable on standardize measures of SIN perception (i.e., QuickSIN test) (Bidelman et al., 2019a). Still, future studies are needed to determine (i) if our brain decoding results generalize to more severe (or even undiagnosed) hearing losses, (ii) scale with individual differences in perceptual skills, and (iii) might change with cognitive decline that is common in older adults (e.g., Bidelman et al., 2017).

### Noise-Degraded Speech Processing Requires More (Right Hemisphere) Neural Resources Than Clear Speech Processing

We extend previous neuroimaging studies by demonstrating the most stable, consistent, and invariant functional brain regions supporting age-related speech and SIN processing using a data-driven approach (stability selection coupled with SVM). Stability selection with randomized Lasso on full-brain neural data identified the most important brain regions associated with hearing loss over a range of model parameters. Our analysis revealed the most stable brain regions could segregate groups at >80% accuracy from the ERP features alone corresponding to an optimal stability score (0.1). Group segregation was still reasonably accurate using a more stringent stability score of 0.5, which identified a sparser subset of brain regions that described age-related declines in speech processing (Table 2).

For clear speech perception, stability selection identified 12 regions, four ROIs from temporal lobe including bilateral superior temporal sulcus, three regions from frontal, and three from parietal lobe. For noise-degraded speech perception, five important regions emerged in the temporal lobe including bilateral superior temporal sulcus, four regions from frontal lobe, four from partial lobe, and one region from occipital lobe. For both clear and noise-degraded speech, a greater number of regions were recruited from the temporal lobe. This finding supports previous studies (Crinion et al., 2003; Okada et al., 2010; Peelle et al., 2010) that suggest a critical engagement of temporal lobe for speech perception.

Among the two networks identified via stability selection, eight regions were common for clear and noise-degraded speech perception. One of these, the right temporal pole, is part of the auditory ventral pathway which plays a role in the coding, representation, and perception of nonspatial attributes of sound including auditory identity (Rauschecker and Tian, 2000; Alain et al., 2009). Recruitment of precentral gyrus in our speech tasks is probably also anticipated given the role of primary motor areas in phoneme processing, particularly in noise (Du et al., 2014; Hickok, 2014). Superior temporal areas – especially in left hemisphere (r/lBKS, lST) – were also recruited, consistent with their role in the phonological network (Skeide and Friederici, 2016).

Interestingly, for noise-degraded speech perception, we found several non-overlapping regions including dorsolateral prefrontal cortex (rostral middle frontal). The additional recruitment of this area when the phonemes were embedded in noise most probably reflects compensatory processing associated with working memory and higher-order speech processing (Zatorre et al., 1992; Gabrieli et al., 1998; Wong et al., 2004; Alain et al., 2018), which would necessarily need to be engaged during the more complex listening demands of noise. The other non-overlapping areas in the inferior parietal lobe (lIP/rIP) are associated with phonological (Burton, 2001) and auditory working memory (Alain et al., 2008). Perhaps involvement of the remaining regions unique to the noise condition also aids noise-degraded speech perception, in a yet unknown way. For example, we speculate that the dual involvement of the pericalcarine and inferior temporal areas in the noise condition may reflect a form of visual or motor imagery listeners use as a strategy to cope with task difficulty (Ganis et al., 2004; Tian and Poeppel, 2012). It is noticeable that for clear speech, about half (6/12 = 50%) of the stable regions were from LH. However, LH involvement was reduced for noisy speech perception (6/16 = 37.50%) which was paralleled by stronger RH involvement (i.e., 10/16 = 62.50% stable regions were right lateralized). This hemisphere asymmetry cannot be explained by differences in hearing between ears as our listeners showed bilateral symmetric audiograms. However, older adults do show symmetric lateralization for clear speech perception, which corroborates the so-called HAROLD model of aging (Cabeza, 2002). On the other hand, older adults showed asymmetric lateralization while perceive noise-degraded speech. Our findings are broadly consistent with previous neuroimaging studies demonstrating that noise-degraded speech perception requires additional RH brain regions to compensate for the impoverished acoustic signal (Shtyrov et al., 1998, 1999; Bidelman and Howell, 2016; Mudar and Husain, 2016).

Collectively, our findings show that additional brain regions are recruited in the temporal, frontal and partial lobe while processing the noise-degraded speech relative to clear speech. Previous neuroimaging studies (Peelle et al., 2009; Wong et al., 2009; Erb and Obleser, 2013; Guediche et al., 2014; Du et al., 2016; Dimitrijevic et al., 2019; Yi et al., 2019) have similarly demonstrated that noise-degraded speech perception increases recruitment of temporal (i.e., perceptual processing), frontal brain (i.e., upregulation of frontal areas), and parietal regions. Our data driven approach corroborates previous studies by confirming more brain regions are allocated to process acoustically degraded compared to clear speech. Nevertheless, given the limited spatial resolution of EEG, future studies using more spatially precise neuroimaging techniques (e.g., fMRI) are needed to fully confirm the stable ROIs observed here.

## Conclusion

We investigated when and where cortical brain activity segregates NH and HI listeners by using multivariate analyses on EEG recordings obtained while the subjects were performing a specific task. The proposed data driven approach showed that the P1 wave of the auditory ERPs robustly distinguish NH and HI groups, revealing speech-evoked neural responses are highly sensitive to age-related hearing loss. Our results further suggest that identifying listeners with mild hearing impairment based on their EEGs is also 3.75% more robust when using LH compared to RH features of brain activity, particularly under listening conditions that tax the auditory system (i.e., noise interference). From stability selection and SVM classifier analyses, we identified sparse (<16 regions) yet highly robust networks that describe older adults’ speech processing. Yet, we found more neural resources are required to distinguish hearing-related declines in speech processing in noise, particularly in the right hemisphere.

## Data Availability Statement

The datasets generated for this study are available on request to the corresponding author.

## Ethics Statement

The studies involving human participants were reviewed and approved by Baycrest Research Ethics Review Board. The patients/participants provided their written informed consent to participate in this study.

## Author Contributions

CA designed the experiments and collected the data. MM, MY, and GB analyzed the data. MM, MY, GB, and CA interpreted results of the experiments. MM, FA, MY, and GB drafted the manuscript. RA-F and FA helped implementation of stability selection. KM developed the brain regions visualization tool. All authors edited and revised the manuscript and approved the final version of manuscript.

## Conflict of Interest

The authors declare that the research was conducted in the absence of any commercial or financial relationships that could be construed as a potential conflict of interest.

## Footnotes

1 Stimuli were presented at a fixed intensity (SPL). While adjusting sensation level (SL) would correct for the (mild) audibility issues in our HI listeners, this runs the risk of introducing loudness recruitment issues that often accompany sensorineural hearing loss. Still, we have found similar noise-related changes in HI listeners’ ERPs when stimuli are presented at equal dB SL (Bidelman et al., 2014), suggesting effects would be comparable to those observed here.

2 Parameters γ and *C* in the SVM used in this study gives a measure of the influence of training data points on decision boundary and a measure of miss-classification tolerance. The first parameter γ comes from the radial basis function kernel (e.g., $K\,\left(x,x^{\prime }\right)=\exp\left(-\frac{{||x-x^{\prime }||}^{2}}{2\,\sigma^{2}}\right)$ or equivalently *K*(*x*,*x*′) = exp ⁡ (-γ || *x* − *x*′ || 2) with a parameter γ) where $\gamma =\frac{1}{2\,\sigma^{2}}$. In this study, the radial basis kernel is used as a transformation function. A larger value of γ implies smaller *σ*, which means that the classifier takes into account the effect of samples closer to the decision boundary. On the other hand, smaller γ means that the classifier considers the effect of samples farther from the decision boundary. The *C* is a parameter of SVM that acts as regularization. It provides the classifier a trade-off between the margin of decision boundary and miss – classification. A larger value of *C* produces a narrower (smaller-margin) hyperplane if that obtains less or no miss-classification. Whereas the smaller value of *C* allows drawing a wider (bigger-margin) hyperplane even if there are some miss-classifications. The optimal value of γ and *C* depends on data, which is why we used a grid search to tune these parameters in our classification model.

3 Randomized lasso is a two-step randomization process: i) randomly select a fraction of samples; ii) randomly select the regularization parameter (to build a new model iteration). This process iterates ‘*N*’ number of times to build *‘N’* number of randomized models. Sample fraction refers to the fraction of samples to be used in each randomized design. The resampling number refers to the number of randomized models.

4 Actual sample data were divided into 80% as training and 20% as the test set. The 80% is divided into 5 equal size chunks. One chunk is left as a validation set during training. The SVM was trained on the remaining 4 chunks and validated with the left a chunk alone. This loop continued 5 times to get SVM trained on the 80% training set and the best estimation is kept as a trained model. We then evaluated the model performance by submitting the features of the 20% unseen test data.

## References

[B1] Agung K. Purdy S. C. McMahon C. M. Newall P. (2006). The use of cortical auditory evoked potentials to evaluate neural encoding of speech sounds in adults. *J. Am. Acad. Audiol.* 17 559–572. 10.3766/jaaa.17.8.3 16999251

[B2] Alain C. (2014). Effects of age-related hearing loss and background noise on neuromagnetic activity from auditory cortex. *Front. Syst. Neurosci.* 8:8. 10.3389/fnsys.2014.00008 24550790 PMC3907769

[B3] Alain C. Du Y. Bernstein L. J. Barten T. Banai K. (2018). Listening under difficult conditions: an activation likelihood estimation meta-analysis. *Hum. Brain Mapp.* 39 2695–2709. 10.1002/hbm.24031 29536592 PMC6866419

[B4] Alain C. He Y. Grady C. (2008). The contribution of the inferior parietal lobe to auditory spatial working memory. *J. Cogn. Neurosci.* 20 285–295. 10.1162/jocn.2008.20014 18275335

[B5] Alain C. McDonald K. L. Kovacevic N. McIntosh A. R. (2009). Spatiotemporal analysis of auditory “what” and “where” working memory. *Cereb. Cortex* 19 305–314. 10.1093/cercor/bhn082 18534993

[B6] Alain C. McDonald K. L. Ostroff J. M. Schneider B. (2004). Aging: a switch from automatic to controlled processing of sounds? *Psychol. Aging* 19 125–133. 10.1037/0882-7974.19.1.125 15065936

[B7] Alain C. Snyder J. S. (2008). Age-related differences in auditory evoked responses during rapid perceptual learning. *Clin. Neurophysiol.* 119 356–366. 10.1016/j.clinph.2007.10.024 18083619

[B8] Al-Fahad R. Yeasin M. Bidelman G. (2019). Decoding of single-trial EEG reveals unique states of functional brain connectivity that drive rapid speech categorization decisions. *J. Neural Eng.* 17:016045. 10.1088/1741-2552/ab6040 31822643 PMC7004853

[B9] Anderson S. Parbery-Clark A. White-Schwoch T. Kraus N. (2012). Aging affects neural precision of speech encoding. *J. Neurosci.* 32 14156–14164. 10.1523/jneurosci.2176-12.2012 23055485 PMC3488287

[B10] Anderson S. Skoe E. Chandrasekaran B. Kraus N. (2010). Neural timing is linked to speech perception in noise. *J. Neurosci.* 30 4922–4926. 10.1523/JNEUROSCI.0107-10.2010 20371812 PMC2862599

[B11] Bhasin M. Raghava G. P. S. (2004). SVM based method for predicting HLA-DRB1^∗^0401 binding peptides in an antigen sequence. *Bioinformatics* 20 421–423. 10.1093/bioinformatics/btg424 14960470

[B12] Bidelman G. M. Howell M. (2016). Functional changes in inter- and intra-hemispheric cortical processing underlying degraded speech perception. *Neuroimage* 124 581–590. 10.1016/j.neuroimage.2015.09.020 26386346

[B13] Bidelman G. M. Lowther J. E. Tak S. H. Alain C. (2017). Mild cognitive impairment is characterized by deficient brainstem and cortical representations of speech. *J. Neurosci.* 37 3610–3620. 10.1523/jneurosci.3700-16.2017 28270574 PMC6596919

[B14] Bidelman G. M. Mahmud M. S. Yeasin M. Shen D. Arnott S. R. Alain C. (2019a). Age-related hearing loss increases full-brain connectivity while reversing directed signaling within the dorsal–ventral pathway for speech. *Brain Struct. Funct.* 224 2661–2676. 10.1007/s00429-019-01922-9 31346715 PMC6778722

[B15] Bidelman G. M. Price C. N. Shen D. Arnott S. R. Alain C. (2019b). Afferent-efferent connectivity between auditory brainstem and cortex accounts for poorer speech-in-noise comprehension in older adults. *Hear. Res.* 382:107795. 10.1016/j.heares.2019.107795 31479953 PMC6778515

[B16] Bidelman G. M. Villafuerte J. W. Moreno S. Alain C. (2014). Age-related changes in the subcortical–cortical encoding and categorical perception of speech. *Neurobiol. Aging* 35 2526–2540. 10.1016/j.neurobiolaging.2014.05.006 24908166

[B17] Bidelman G. M. Walker B. (2019). Plasticity in auditory categorization is supported by differential engagement of the auditory-linguistic network. *NeuroImage* 201:116022. 10.1016/j.neuroimage.2019.116022 31310863 PMC6765438

[B18] Bidelman G. M. Yellamsetty A. (2017). Noise and pitch interact during the cortical segregation of concurrent speech. *Hear. Res.* 351 34–44. 10.1016/j.heares.2017.05.008 28578876

[B19] Billings C. J. McMillan G. P. Penman T. M. Gille S. M. (2013). Predicting perception in noise using cortical auditory evoked potentials. *J. Assoc. Res. Otolaryngol.* 14 891–903. 10.1007/s10162-013-0415-y 24030818 PMC3825022

[B20] Billings C. J. Penman T. M. McMillan G. P. Ellis E. M. (2015). Electrophysiology and perception of speech in noise in older listeners: effects of hearing impairment and age. *Ear Hear.* 36 710–722. 10.1097/AUD.0000000000000191 26502191 PMC4621778

[B21] Blackwell D. L. Lucas J. W. Clarke T. C. (2014). Summary health statistics for U.S. adults: national health interview survey, 2012. *Vital Health Stat.* 10 1–161. 24819891

[B22] Brette R. (2012). Computing with neural synchrony. *PLoS Computat. Biol.* 8:e1002561. 10.1371/journal.pcbi.1002561 22719243 PMC3375225

[B23] Burkard R. F. Sims D. (2002). A comparison of the effects of broadband masking noise on the auditory brainstem response in young and older adults. *Am. J. Audiol.* 11 13–22. 10.1044/1059-0889(2002/004) 12227352

[B24] Burton M. W. (2001). The role of inferior frontal cortex in phonological processing. *Cogn. Sci.* 25 695–709. 10.1207/s15516709cog2505_4

[B25] Cabeza R. (2002). Hemispheric asymmetry reduction in older adults: the HAROLD model. *Psychol. Aging* 17:85. 10.1037/0882-7974.17.1.85 11931290

[B26] Casale S. Russo A. Scebba G. Serrano S. (2008). “Speech Emotion Classification Using Machine Learning Algorithms,” in *Proceedings of the 2008 IEEE International Conference on Semantic Computing*, Santa Clara, CA, 158–165.

[B27] Caspary D. M. Hughes L. F. Schatteman T. A. Turner J. G. (2006). Age-related changes in the response properties of cartwheel cells in rat dorsal cochlear nucleus. *Hear. Res.* 216 207–215. 10.1016/j.heares.2006.03.005 16644158

[B28] Caspary D. M. Ling L. Turner J. G. Hughes L. F. (2008). Inhibitory neurotransmission, plasticity and aging in the mammalian central auditory system. *J. Exp. Biol.* 211 1781–1791. 10.1242/jeb.013581 18490394 PMC2409121

[B29] Crinion J. T. Lambon-Ralph M. A. Warburton E. A. Howard D. Wise R. J. (2003). Temporal lobe regions engaged during normal speech comprehension. *Brain* 126 1193–1201. 10.1093/brain/awg104 12690058

[B30] Cruickshanks K. J. Wiley T. L. Tweed T. S. Klein B. E. Klein R. Mares-Perlman J. A. (1998). Prevalence of hearing loss in older adults in Beaver Dam, Wisconsin: the epidemiology of hearing loss study. *Am. J. Epidemiol.* 148 879–886. 10.1093/oxfordjournals.aje.a009713 9801018

[B31] Desikan R. S. Ségonne F. Fischl B. Quinn B. T. Dickerson B. C. Blacker D. (2006). An automated labeling system for subdividing the human cerebral cortex on MRI scans into gyral based regions of interest. *NeuroImage* 31 968–980. 10.1016/j.neuroimage.2006.01.021 16530430

[B32] Diaz M. T. Johnson M. A. Burke D. M. Truong T.-K. Madden D. J. (2018). Age-related differences in the neural bases of phonological and semantic processes in the context of task-irrelevant information. *Cogn. Affect. Behav. Neurosci.* 19 829–844. 10.3758/s13415-018-00671-2 30488226 PMC6538491

[B33] Dimitrijevic A. Smith M. L. Kadis D. S. Moore D. R. (2019). Neural indices of listening effort in noisy environments. *Sci. Rep.* 9 1–10. 31375712 10.1038/s41598-019-47643-1 PMC6677804

[B34] Ding N. Simon J. Z. (2013). Adaptive temporal encoding leads to a background-insensitive cortical representation of speech. *J. Neurosci.* 33 5728–5735. 10.1523/JNEUROSCI.5297-12.2013 23536086 PMC3643795

[B35] Du Y. Buchsbaum B. R. Grady C. L. Alain C. (2014). Noise differentially impacts phoneme representations in the auditory and speech motor systems. *Proc. Natl. Acad. Sci. U.S.A.* 111 7126–7131. 10.1073/pnas.1318738111 24778251 PMC4024897

[B36] Du Y. Buchsbaum B. R. Grady C. L. Alain C. (2016). Increased activity in frontal motor cortex compensates impaired speech perception in older adults. *Nat. Commun.* 7:12241. 10.1038/ncomms12241 27483187 PMC4974649

[B37] Dubno J. R. Schaefer A. B. (1992). Comparison of frequency selectivity and consonant recognition among hearing-impaired and masked normal-hearing listeners. *J. Acoust. Soc. Am.* 91 2110–2121. 10.1121/1.403697 1597602

[B38] Efron B. Hastie T. Johnstone I. Tibshirani R. (2004). Least angle regression. *Ann. Stat.* 32 407–499.

[B39] Eggermont J. J. Ponton C. W. Don M. Waring M. D. Kwong B. (1997). Maturational delays in cortical evoked potentials in cochlear implant users. *Acta Otolaryngol.* 117 161–163. 10.3109/00016489709117760 9105439

[B40] Erb J. Obleser J. (2013). Upregulation of cognitive control networks in older adults’ speech comprehension. *Front. Syst. Neurosci.* 7:116. 10.3389/fnsys.2013.00116 24399939 PMC3871967

[B41] Erwin R. J. Buchwald J. S. (1987). Midlatency auditory evoked responses in the human and the cat model. *Electroencephalogr. Clin. Neurophysiol.* 40 461–467. 3480164

[B42] Friedman J. Hastie T. Tibshirani R. (2010). Regularization paths for generalized linear models via coordinate descent. *J. Stat. Softw.* 33 1–22. 20808728 PMC2929880

[B43] Frost J. A. Binder J. R. Springer J. A. Hammeke T. A. Bellgowan P. S. Rao S. M. (1999). Language processing is strongly left lateralized in both sexes: evidence from functional MRI. *Brain* 122 199–208. 10.1093/brain/122.2.199 10071049

[B44] Fuchs M. Drenckhahn R. Wischmann H. A. Wagner M. (1998). An improved boundary element method for realistic volume-conductor modeling. *IEEE Trans. Biomed. Eng.* 45 980–997. 10.1109/10.704867 9691573

[B45] Fuchs M. Kastner J. Wagner M. Hawes S. Ebersole J. S. (2002). A standardized boundary element method volume conductor model. *Clin. Neurophysiol.* 113 702–712. 10.1016/s1388-2457(02)00030-5 11976050

[B46] Furey T. S. Cristianini N. Duffy N. Bednarski D. W. Schummer M. Haussler D. (2000). Support vector machine classification and validation of cancer tissue samples using microarray expression data. *Bioinformatics* 16 906–914. 10.1093/bioinformatics/16.10.906 11120680

[B47] Gabrieli J. D. Poldrack R. A. Desmond J. E. (1998). The role of left prefrontal cortex in language and memory. *Proc. Natl. Acad. Sci. U.S.A.* 95 906–913. 9448258 10.1073/pnas.95.3.906 PMC33815

[B48] Ganis G. Thompson W. L. Kosslyn S. M. (2004). Brain areas underlying visual mental imagery and visual perception: an fMRI study. *Cogn. Brain Res.* 20 226–241. 10.1016/j.cogbrainres.2004.02.012 15183394

[B49] Gates G. A. Mills J. H. (2005). Presbycusis. *Lancet* 366 1111–1120. 16182900 10.1016/S0140-6736(05)67423-5

[B50] Gordon-Salant S. Fitzgibbons P. J. (1993). Temporal factors and speech recognition performance in young and elderly listeners. *J. Speech Lang. Hear Res.* 36 1276–1285. 10.1044/jshr.3606.1276 8114494

[B51] Grady C. L. (2008). Cognitive neuroscience of aging. *Ann. N. Y. Acad. Sci.* 1124 127–144. 18400928 10.1196/annals.1440.009

[B52] Gramfort A. Papadopoulo T. Olivi E. Clerc M. (2010). OpenMEEG: opensource software for quasistatic bioelectromagnetics. *Biomed. Eng.* 9:45. 10.1186/1475-925X-9-45 20819204 PMC2949879

[B53] Guediche S. Blumstein S. Fiez J. Holt L. L. (2014). Speech perception under adverse conditions: insights from behavioral, computational, and neuroscience research. *Front. Syst. Neurosci.* 7:126. 10.3389/fnsys.2013.00126 24427119 PMC3879477

[B54] Hickok G. (2014). The architecture of speech production and the role of the phoneme in speech processing. *Lang. Cogn. Neurosci.* 29 2–20. 10.1080/01690965.2013.834370 24489420 PMC3904400

[B55] Hickok G. Poeppel D. (2004). Dorsal and ventral streams: a framework for understanding aspects of the functional anatomy of language. *Cognition* 92 67–99. 10.1016/j.cognition.2003.10.011 15037127

[B56] Hickok G. Poeppel D. (2007). The cortical organization of speech processing. *Nat. Rev. Neurosci.* 8:393. 10.1038/nrn2113 17431404

[B57] Hsu C.-W. Chang C.-C. Lin C. J. (2003). *A Practical Guide to Support Vector Classification Technical Report Department of Computer Science and Information Engineering.* Taipei: National Taiwan University.

[B58] Humes L. E. Busey T. A. Craig J. Kewley-Port D. (2013). Are age-related changes in cognitive function driven by age-related changes in sensory processing? *Attent. Percept. Psychophys.* 75 508–524. 10.3758/s13414-012-0406-9 23254452 PMC3617348

[B59] Humes L. E. Dubno J. R. Gordon-Salant S. Lister J. J. Cacace A. T. Cruickshanks K. J. (2012). Central presbycusis: a review and evaluation of the evidence. *J. Am. Acad. Audiol.* 23 635–666. 10.3766/jaaa.23.8.5 22967738 PMC5898229

[B60] Hutka S. A. Alain C. Binns M. A. Bidelman G. M. (2013). Age-related differences in the sequential organization of speech sounds. *J. Acoust. Soc. Am.* 133 4177–4187. 10.1121/1.4802745 23742369

[B61] Jang J. H. Jang H. K. Kim S. E. Oh S. H. Chang S. O. Lee J. H. (2010). Analysis of P1 latency in normal hearing and profound sensorineural hearing loss. *Clin. Exp. Otorhinolaryngol.* 3 194–198. 10.3342/ceo.2010.3.4.194 21217959 PMC3010537

[B62] Killion M. C. Niquette P. A. Gudmundsen G. I. Revit L. J. Banerjee S. (2004). Development of a quick speech-in-noise test for measuring signal-to-noise ratio loss in normal-hearing and hearing-impaired listeners. *J. Acoust. Soc. Am.* 116 2395–2405. 10.1121/1.1784440 15532670

[B63] Kim J.-R. Ahn S.-Y. Jeong S.-W. Kim L.-S. Park J.-S. Chung S.-H. (2012). Cortical auditory evoked potential in aging: effects of stimulus intensity and noise. *Otol. Neurotol.* 33 1105–1112. 10.1097/mao.0b013e3182659b1e 22892802

[B64] Koerner T. K. Zhang Y. (2015). Effects of background noise on inter-trial phase coherence and auditory N1–P2 responses to speech stimuli. *Hear. Res.* 328 113–119. 10.1016/j.heares.2015.08.002 26276419

[B65] Konkle D. F. Beasley D. S. Bess F. H. (1977). Intelligibility of time-altered speech in relation to chronological aging. *J. Speech Hear. Res.* 20 108–115. 10.1044/jshr.2001.108 846194

[B66] Konrad-Martin D. Dille M. F. McMillan G. Griest S. McDermott D. Fausti S. A. (2012). Age-related changes in the auditory brainstem response. *J. Am. Acad. Audiol.* 23 18–35. 22284838 10.3766/jaaa.23.1.3 PMC5549623

[B67] Kujawa S. G. Liberman M. C. (2015). Synaptopathy in the noise-exposed and aging cochlea: primary neural degeneration in acquired sensorineural hearing loss. *Hear. Res.* 330 191–199. 10.1016/j.heares.2015.02.009 25769437 PMC4567542

[B68] Liberman M. C. (2017). Noise-induced and age-related hearing loss: new perspectives and potential therapies. *F1000Research* 6:927. 10.12688/f1000research.11310.1 28690836 PMC5482333

[B69] Liegeois-Chauvel C. Musolino A. Badier J. M. Marquis P. Chauvel P. (1994). Evoked potentials recorded from the auditory cortex in man: evaluation and topography of the middle latency components. *Electroencephalogr. Clin. Neurophysiol.* 92 204–214. 10.1016/0168-5597(94)90064-7 7514990

[B70] Lin F. R. Yaffe K. Xia J. Xue Q.-L. Harris T. B. Purchase-Helzner E. (2013). Hearing loss and cognitive decline in older adults. *J. Am. Med. Assoc.* 173 293–299. 10.1001/jamainternmed.2013.1868 PMC3869227 23337978

[B71] Mahmud M. S. Yeasin M. Shen D. Arnott S. R. Alain C. Bidelman G. M. (2018). “What brain connectivity patterns from EEG tell us about hearing loss: a graph theoretic approach,” in *Proceedings of the 2018 10th International Conference on Electrical and Computer Engineering (ICECE)* (Piscataway, NJ: IEEE), 205–208.

[B72] Mazziotta J. C. Toga A. W. Evans A. Fox P. Lancaster J. (1995). A probabilistic atlas of the human brain: theory and rationale for its development. The International Consortium for Brain Mapping (ICBM). *Neuroimage* 2 89–101. 10.1006/nimg.1995.1012 9343592

[B73] McGee T. Kraus N. (1996). Auditory development reflected by middle latency response. *Ear Hear.* 17 419–429. 10.1097/00003446-199610000-00008 8909890

[B74] Meinshausen N. Bühlmann P. (2010). Stability selection. *J. R. Stat. Soc. Ser. B* 72 417–473. 10.1111/j.1467-9868.2010.00740.x

[B75] Michel C. M. Murray M. M. Lantz G. Gonzalez S. Spinelli L. Grave de Peralta R. (2004). EEG source imaging. *Clin. Neurophysiol.* 115 2195–2222. 10.1016/j.clinph.2004.06.001 15351361

[B76] Moinuddin K. A. Yeasin M. Bidelman G. M. (2019). *BrainO.* Available online at: https://github.com/cvpia-uofm/BrainO (accessed September 9, 2019).

[B77] Mudar R. A. Husain F. T. (2016). Neural alterations in acquired age-related hearing loss. *Front. Psychol.* 7:828. 10.3389/fpsyg.2016.00828 27313556 PMC4889579

[B78] Murray C. J. Abraham J. Ali M. K. Alvarado M. Atkinson C. Baddour L. M. (2013). The state of US health, 1990-2010: burden of diseases, injuries, and risk factors. *J. Am. Med. Assoc.* 310 591–606. 10.1001/jama.2013.13805 PMC5436627 23842577

[B79] Nogueira S. Sechidis K. Brown G. (2017). On the stability of feature selection algorithms. *J. Mach. Learn. Res.* 18 1–54.

[B80] Okada K. Rong F. Venezia J. Matchin W. Hsieh I.-H. Saberi K. (2010). Hierarchical organization of human auditory cortex: evidence from acoustic invariance in the response to intelligible speech. *Cereb. Cortex* 20 2486–2495. 10.1093/cercor/bhp318 20100898 PMC2936804

[B81] Oostenveld R. Praamstra P. (2001). The five percent electrode system for high-resolution EEG and ERP measurements. *Clin. Neurophysiol.* 112 713–719. 10.1016/s1388-2457(00)00527-7 11275545

[B82] Park Y. Luo L. Parhi K. K. Netoff T. (2011). Seizure prediction with spectral power of EEG using cost-sensitive support vector machines. *Epilepsia* 52 1761–1770. 10.1111/j.1528-1167.2011.03138.x 21692794

[B83] Pascual-Marqui R. D. Esslen M. Kochi K. Lehmann D. (2002). Functional imaging with low-resolution brain electromagnetic tomography (LORETA): a review. *Methods Find. Exp. Clin. Pharmacol.* 24 (Suppl. C), 91–95. 12575492

[B84] Peelle J. E. Johnsrude I. Davis M. H. (2010). Hierarchical processing for speech in human auditory cortex and beyond. *Front. Hum. Neurosci.* 4:51. 10.3389/fnhum.2010.00051 20661456 PMC2907234

[B85] Peelle J. E. Troiani V. Wingfield A. Grossman M. (2009). Neural processing during older adults’ comprehension of spoken sentences: age differences in resource allocation and connectivity. *Cereb. Cortex* 20 773–782. 10.1093/cercor/bhp142 19666829 PMC2837088

[B86] Peelle J. E. Wingfield A. (2016). The neural consequences of age-related hearing loss. *Trends Neurosci.* 39 486–497. 10.1016/j.tins.2016.05.001 27262177 PMC4930712

[B87] Picton T. W. Alain C. Woods D. L. John M. S. Scherg M. Valdes-Sosa P. (1999). Intracerebral sources of human auditory-evoked potentials. *Audiol. Neurootol.* 4 64–79. 10.1159/000013823 9892757

[B88] Picton T. W. van Roon P. Armilio M. L. Berg P. Ille N. Scherg M. (2000). The correction of ocular artifacts: a topographic perspective. *Clin. Neurophysiol.* 111 53–65. 10.1016/S1388-2457(99)00227-8 10656511

[B89] Polat K. Güneş S. (2007). Breast cancer diagnosis using least square support vector machine. *Digital Signal Process.* 17 694–701. 10.1016/j.dsp.2006.10.008

[B90] Presacco A. Simon J. Z. Anderson S. (2016). Effect of informational content of noise on speech representation in the aging midbrain and cortex. *J. Neurophysiol.* 116 2356–2367. 10.1152/jn.00373.2016 27605531 PMC5110638

[B91] Price C. N. Alain C. Bidelman G. M. (2019). Auditory-frontal channeling in α and β bands is altered by age-related hearing loss and relates to speech perception in noise. *Neuroscience* 423 18–28. 10.1016/j.neuroscience.2019.10.044 31705894 PMC6900454

[B92] Rauschecker J. P. Scott S. K. (2009). Maps and streams in the auditory cortex: nonhuman primates illuminate human speech processing. *Nat. Neurosci.* 12:718. 10.1038/nn.2331 19471271 PMC2846110

[B93] Rauschecker J. P. Tian B. (2000). Mechanisms and streams for processing of “what” and “where” in auditory cortex. *Proc. Natl. Acad. Sci. U.S.A.* 97 11800–11806. 10.1073/pnas.97.22.11800 11050212 PMC34352

[B94] Roque L. Karawani H. Gordon-Salant S. Anderson S. (2019). Effects of age, cognition, and neural encoding on the perception of temporal speech cues. *Front. Neurosci.* 13:749. 10.3389/fnins.2019.00749 31379494 PMC6659127

[B95] Ross B. Snyder J. S. Aalto M. McDonald K. L. Dyson B. J. Schneider B. (2009). Neural encoding of sound duration persists in older adults. *Neuroimage* 47 678–687. 10.1016/j.neuroimage.2009.04.051 19393323

[B96] Saito T. Rehmsmeier M. (2015). The precision-recall plot is more informative than the ROC plot when evaluating binary classifiers on imbalanced datasets. *PLoS One* 10:e0118432. 10.1371/journal.pone.0118432 25738806 PMC4349800

[B97] Schneider B. A. Daneman M. Pichora-Fuller M. K. (2002). Listening in aging adults: from discourse comprehension to psychoacoustics. *Can. J. Exp. Psychol.* 56 139–152. 10.1037/h0087392 12271745

[B98] Schoof T. Rosen S. (2016). The role of age-related declines in subcortical auditory processing in speech perception in noise. *J. Assoc. Res. Otolaryngol.* 17 441–460. 10.1007/s10162-016-0564-x 27216166 PMC5023535

[B99] Shtyrov Y. Kujala T. Ahveninen J. Tervaniemi M. Alku P. Ilmoniemi R. J. (1998). Background acoustic noise and the hemispheric lateralization of speech processing in the human brain: magnetic mismatch negativity study. *Neurosci. Lett.* 251 141–144. 10.1016/s0304-3940(98)00529-1 9718994

[B100] Shtyrov Y. Kujala T. Ilmoniemi R. J. Näätänen R. (1999). Noise affects speech-signal processing differently in the cerebral hemispheres. *NeuroReport* 10 2189–2192. 10.1097/00001756-199907130-00034 10424696

[B101] Skeide M. A. Friederici A. D. (2016). The ontogeny of the cortical language network. *Nat. Rev. Neurosci.* 17 323. 10.1038/nrn.2016.23 27040907

[B102] Snyder J. S. Alain C. (2005). Age-related changes in neural activity associated with concurrent vowel segregation. *Cogn. Brain Res.* 24 492–499. 10.1016/j.cogbrainres.2005.03.002 16099361

[B103] Song J. Davey C. Poulsen C. Luu P. Turovets S. Anderson E. (2015). EEG source localization: sensor density and head surface coverage. *J. Neurosci. Methods* 256 9–21. 10.1016/j.jneumeth.2015.08.015 26300183

[B104] Strouse A. Ashmead D. H. Ohde R. N. Grantham D. W. (1998). Temporal processing in the aging auditory system. *J. Acoust. Soc. Am.* 104 2385–2399. 10491702 10.1121/1.423748

[B105] Tadel F. Baillet S. Mosher J. C. Pantazis D. Leahy R. M. (2011). Brainstorm: a user-friendly application for MEG/EEG analysis. *Comput. Intellig. Neurosci.* 2011:879716. 10.1155/2011/879716 PMC3090754 21584256

[B106] Tervaniemi M. Hugdahl K. (2003). Lateralization of auditory-cortex functions. *Brain Res. Rev.* 43 231–246. 10.1016/j.brainresrev.2003.08.004 14629926

[B107] Tian X. Poeppel D. (2012). Mental imagery of speech: linking motor and perceptual systems through internal simulation and estimation. *Front. Hum. Neurosci.* 6:314. 10.3389/fnhum.2012.00314 23226121 PMC3508402

[B108] Tremblay K. Kraus N. McGee T. Ponton C. Otis B. (2001). Central auditory plasticity: changes in the N1-P2 complex after speech-sound training. *Ear Hear.* 22 79–90. 10.1097/00003446-200104000-00001 11324846

[B109] Tremblay K. L. Piskosz M. Souza P. (2003). Effects of age and age-related hearing loss on the neural representation of speech cues. *Clin. Neurophysiol.* 114 1332–1343. 10.1016/s1388-2457(03)00114-7 12842732

[B110] Vaden K. I. Kuchinsky S. E. Ahlstrom J. B. Dubno J. R. Eckert M. A. (2015). Cortical activity predicts which older adults recognize speech in noise and when. *J. Neurosci.* 35 3929–3937. 10.1523/jneurosci.2908-14.2015 25740521 PMC4348188

[B111] van Rooij J. C. Plomp R. (1992). Auditive and cognitive factors in speech perception by elderly listeners. III. Additional data and final discussion. *J. Acoust. Soc. Am.* 91 1028–1033. 10.1121/1.402628 1556304

[B112] Vos T. Barber R. M. Bell B. Bertozzi-Villa A. Biryukov S. Bolliger I. (2015). Global, regional, and national incidence, prevalence, and years lived with disability for 301 acute and chronic diseases and injuries in 188 countries, 1990–2013: a systematic analysis for the Global Burden of Disease Study 2013. *Lancet* 386 743–800. 10.1016/S0140-6736(15)60692-4 26063472 PMC4561509

[B113] Wong P. C. Jin J. X. Gunasekera G. M. Abel R. Lee E. R. Dhar S. (2009). Aging and cortical mechanisms of speech perception in noise. *Neuropsychologia* 47 693–703. 10.1016/j.neuropsychologia.2008.11.032 19124032 PMC2649004

[B114] Wong P. C. Parsons L. M. Martinez M. Diehl R. L. (2004). The role of the insular cortex in pitch pattern perception: the effect of linguistic contexts. *J. Neurosci.* 24 9153–9160. 10.1523/jneurosci.2225-04.2004 15483134 PMC6730056

[B115] Woods D. L. Clayworth C. C. (1986). Age-related changes in human middle latency auditory evoked potentials. *Electroencephalogr. Clin. Neurophysiol.* 65 297–303. 10.1016/0168-5597(86)90008-0 2424742

[B116] Yi H. G. Leonard M. K. Chang E. F. (2019). The encoding of speech sounds in the superior temporal gyrus. *Neuron* 102 1096–1110. 10.1016/j.neuron.2019.04.023 31220442 PMC6602075

[B117] Yin Q.-Y. Li J.-L. Zhang C.-X. (2017). Ensembling Variable Selectors by Stability Selection for the Cox Model. *Comput. Intellig. Neurosci.* 2017:2747431. 10.1155/2017/2747431 29270195 PMC5706076

[B118] Zatorre R. J. Evans A. C. Meyer E. Gjedde A. (1992). Lateralization of phonetic and pitch discrimination in speech processing. *Science* 256 846–849. 10.1126/science.1589767 1589767

[B119] Zendel B. R. Alain C. (2014). Enhanced attention-dependent activity in the auditory cortex of older musicians. *Neurobiol. Aging* 35 55–63. 10.1016/j.neurobiolaging.2013.06.022 23910654

